# The influence of the geroprotective cytokine on the transcriptome of young and senescent mesenchymal stem cells

**DOI:** 10.1186/s13104-025-07262-8

**Published:** 2025-04-24

**Authors:** Liya G. Kondratyeva, Diana K. Matveeva, Maria I. Ezdakova, Marina V. Utkina, Andrey Yu. Ratushnyy

**Affiliations:** 1 Shemyakin-Ovchinnikov Institute of Bioorganic Chemistry of the Russian Academy of Sciences, Miklukho-Maklaya, 16/10, Moscow, 117997 Russia; 2 Institute of Biomedical Problems, Russian Academy of Sciences, Khoroshevskoye Shosse, 76a, Moscow, 123007 Russia; 3 Department of General, Molecular and Population Genetics, Endocrinology Research Centre, Dm. Ulyanova St., 11, Moscow, 117292 Russia

**Keywords:** RNA-sequencing, Transcriptome, Mesenchymal stem cells, MSCs, Cell senescence, GDF11, Geroprotectors

## Abstract

**Objectives:**

Increasing longevity and the growing elderly population necessitate a deeper understanding of aging mechanisms to prolong productive life and improve treatments for age-related diseases linked with cellular senescence. Mesenchymal stem cells (MSCs) are crucial for maintaining tissue homeostasis, but their physiological changes during senescence are not well understood. Growth differentiation factor 11 (GDF11) has emerged as a potential rejuvenation factor, enhancing MSC viability, mobility, and angiogenic functions, which improves outcomes in ischemic models and cardiac repair. This study aims to identify transcriptomic changes in young and senescent MSCs influenced by GDF11, highlighting its potential in MSC-based therapies.

**Data description:**

To evaluate transcriptomic changes induced by the potential geroprotective factor GDF11, we performed RNA sequencing on four groups of samples: ‘young’ MSCs (MmC-/GDF11-) and senescent MSCs (MmC+/GDF11-) without the addition of GDF11, as well as ‘young’ (MmC-/GDF11+) and senescent MSCs (MmC+/GDF11+) with the addition of GDF11. After 10 days of incubation, indexed cDNA libraries for Illumina sequencing were prepared from the samples, and the resulting cDNA library mix was subjected to NovaSeq 6000 sequencing. This paper describes the collection of 16 RNA sequencing samples comprising 4 sets of MSCs. FASTQ files from Illumina sequencing are available in the NCBI Gene Expression Omnibus.

## Objective

Increasing longevity and the rapid growth in the number of elderly people highlight the need to prolong the productive period of life. This requires a deeper understanding of the fundamental mechanisms of aging to find ways to regulate them and develop better approaches for treating age-related pathologies. To date, various influences on key cellular and molecular mechanisms associated with aging have been actively studied. These approaches aim to suppress pathological processes and activate the defense systems of both the cell and the organism. In this context, the study of mesenchymal stem cells (MSCs) is especially important. MSCs play a crucial role in the aging process, as their primary function is to maintain tissue homeostasis, which changes with age. However, our understanding of the changes in MSC physiology upon senescence activation remains insufficient. The search for ways to modulate the regulatory functions of MSCs is extremely crucial. Paracrine mediators with geroprotective properties are currently of great interest in this field.

Growth differentiation factor 11 (GDF11) has emerged as a potential rejuvenation factor with significant effects on age-related diseases and organ regeneration [1, 2]. It enhances the therapeutic potential of MSCs by improving their viability, mobility, and angiogenic paracrine functions, which leads to better outcomes in ischemic hindlimb models [3]. GDF11 also promotes MSC survival and retention in infarcted hearts, enhancing angiogenesis and cardiac function [4], stimulates MSC differentiation into endothelial-like cells, boosting pro-angiogenic activities [5, 6]. In bone marrow MSCs, GDF11 promotes osteoblastogenesis while inhibiting adipogenesis by altering gene expression patterns and inhibiting PPARγ activity [5]. These effects are mediated through various signaling pathways, including TGF-β receptor, PI3K/Akt, and Smad2/3 [3, 4]. Overall, GDF11 shows promise for enhancing MSC-based therapies in ischemic diseases, cardiac repair, and bone homeostasis. The aim of this study was to identify transcriptomic changes occurring in young and senescent MSCs under the influence of the geroprotective factor GDF11.

## Data description

Experiments were conducted using immortalized standard line of MSCs ASC52telo (ATCC^®^ SCRC-4000™). The DNA alkylating agent mitomycin C (MmC, Sigma Aldrich, St. Louis, MO, USA) was used to produce senescent MSCs (18 h exposition, 1.5 µg/mL in growth medium). Detailed procedures of obtaining senescent MSCs and confirmation of the phenotype were described in [7]. During the following 10 days after MmC treatment, recombinant GDF11 protein (ab218080, Abcam, MA, USA) was added to both senescent and intact MSCs to final concentration 10 ng/mL. MSC without MmC or without GDF11 served as the control. As a result, the study was conducted in four groups: “young” MSCs (MmC-/GDF11-) and senescent (MmC+/GDF11-) cells without the addition of GDF11, as well as “young” (MmC-/GDF11+) and senescent (MmC+/GDF11+) cells with the addition of GDF11. After 10 days of incubation, the cells from each group (with four biological replicates for each group) were collected and subsequently used for total RNA extraction. Total RNA was isolated from MSCs by phenol-chloroform extraction using the ExtractRNA reagent (Evrogen, Russia), followed by treatment with DNase I (Qiagen, MD, USA). The RNA quality was assessed via agarose gel electrophoresis using Agilent TapeStation 4200 System (Agilent Technologies, CA, USA). RNA quality was assessed using the Agilent 2200 TapeStation automated electrophoresis system, and the RNA Integrity Number (RIN) was evaluated. Samples with an RIN > 7 were considered suitable for cDNA library preparation. mRNA-enriched fractions were obtained using the NEBNext^®^ Poly(A) mRNA Magnetic Isolation Module (New England Biolabs, Ipswich, MA, USA) with magnetic beads. For cDNA library construction, the “NEBNext Ultra II Directional RNA Library Prep Kit for Illumina” (NEB) was used in conjunction with the “NEBNext Multiplex Oligos for Illumina.” As a result, 16 amplified indexed cDNA libraries were generated through PCR. The positioning of the adapters introduced at the ends of the fragments of these libraries clearly determined the directionality of the transcript. Finally, the resulting 16 individually indexed RNA-seq libraries (4 × MmC-/GDF11-, 4 × MmC+/GDF11-, 4 × MmC-/GDF11+, 4 × MmC+/GDF11+) were mixed in equimolar amounts, the final mixture was analyzed with Agilent TapeStation system. The median fragment length of the pooled fragments was 319 b.p., distributed between 200 and 700 b.p.

The resulting RNA-seq library mixture was further subjected to NovaSeq 6000 (Illumina) sequencing. Overall, 3,364,706,430 double-end 150 b.p. reads were obtained. The fastq files were analyzed with FASTQC tool [8], the resulting report indicated acceptable sequencing quality for all 16 samples. The mean amount of paired-end reads per library was 105 million. Reads were mapped to the reference genome (GRCh38/hg38) using HISAT2 v2.2.1 [9]. SAM files were converted to BAM format, followed by sorting and indexing using SAMtools v1.9 [10]. The successfully aligned reads were then counted with featureCount tool v.2.0.1 [11] with (GENCODE V38 was used as the reference transcriptome). The final 16 data sets of raw FASTQ data and FeatureCount table were deposited in the NCBI Gene Expression Omnibus (accessible on https://identifiers.org/geo:GSE287646) and listed in Table 1.

**Table 1:** Overview of data files/data sets

Label	Name of data file/data set	File types (file extension)	Data repository and identifier (DOI or accession number)
Data files 1	Raw sequence reads for control MSCs MmC-/GDF11-	FASTQ files (.fastq.gz)	NCBI GEO; http://identifiers.org/geo:GSM8749322 [12]
Data files 2	Raw sequence reads for control MSCs MmC-/GDF11-	FASTQ files (.fastq.gz)	NCBI GEO; http://identifiers.org/geo:GSM8749326 [13]
Data files 3	Raw sequence reads for control MSCs MmC-/GDF11-	FASTQ files (.fastq.gz)	NCBI GEO; http://identifiers.org/geo:GSM8749330 [14]
Data files 4	Raw sequence reads for control MSCs MmC-/GDF11-	FASTQ files (.fastq.gz)	NCBI GEO; https://identifiers.org/geo:GSM8749334 [15]
Data files 5	Raw sequence reads for senescent MSCs MmC+/GDF11-	FASTQ files (.fastq.gz)	NCBI GEO; https://identifiers.org/geo:GSM8749324 [16]
Data files 6	Raw sequence reads for senescent MSCs MmC+/GDF11-	FASTQ files (.fastq.gz)	NCBI GEO; https://identifiers.org/geo:GSM8749328 [17]
Data files 7	Raw sequence reads for senescent MSCs MmC+/GDF11-	FASTQ files (.fastq.gz)	NCBI GEO; https://identifiers.org/geo:GSM8749332 [18]
Data files 8	Raw sequence reads for senescent MSCs MmC+/GDF11-	FASTQ files (.fastq.gz)	NCBI GEO; https://identifiers.org/geo:GSM8749336 [19]
Data files 9	Raw sequence reads for MSCs treated with GDF11 MmC-/GDF11+	FASTQ files (.fastq.gz)	NCBI GEO; https://identifiers.org/geo:GSM8749323 [20]
Data files 10	Raw sequence reads for MSCs treated with GDF11 MmC-/GDF11+	FASTQ files (.fastq.gz)	NCBI GEO; https://identifiers.org/geo:GSM8749327 [21]
Data files 11	Raw sequence reads for MSCs treated with GDF11 MmC-/GDF11+	FASTQ files (.fastq.gz)	NCBI GEO; https://identifiers.org/geo:GSM8749331 [22]
Data files 12	Raw sequence reads for MSCs treated with GDF11 MmC-/GDF11+	FASTQ files (.fastq.gz)	NCBI GEO; https://identifiers.org/geo:GSM8749335 [23]
Data files 13	Raw sequence reads for senescent MSCs treated with GDF11 MmC+/GDF11+	FASTQ files (.fastq.gz)	NCBI GEO; https://identifiers.org/geo:GSM87493325 [24]
Data files 14	Raw sequence reads for senescent MSCs treated with GDF11 MmC+/GDF11+	FASTQ files (.fastq.gz)	NCBI GEO; https://identifiers.org/geo:GSM8749329 [25]
Data files 15	Raw sequence reads for senescent MSCs treated with GDF11 MmC+/GDF11+	FASTQ files (.fastq.gz)	NCBI GEO; https://identifiers.org/geo:GSM8749333 [26]
Data files 16	Raw sequence reads for senescent MSCs treated with GDF11 MmC+/GDF11+	FASTQ files (.fastq.gz)	NCBI GEO; https://identifiers.org/geo:GSM8749337 [27]
Data file 17	FeatureCount table	Table (.txt.gz)	NCBI GEO; http://identifiers.org/geo:GSE287646 [28]

## Limitations

This study is limited by the small sample size, and technical issues resulting in batch effects further reducing the statistical power. Another limitation is conducting experiments using an in vitro MSC cell line model, which may differ from the changes that could be observed at the organism level. MSCs are known to be a heterogeneous population with varying differentiation potentials and gene expression profiles. Bulk RNA-seq averages the gene expression across all cells, potentially masking important differences between subpopulations of MSCs.

## Data Availability

The raw RNA sequencing results for this publication are available from the NCBI Gene Expression Omnibus and are accessible through GEO Series accession number GSE287646 (http://identifiers.org/geo:GSE287646, [28]). The featureCounts table is also available through GEO accession number GSE287646.

## Abbreviations

GDF11: Growth differentiation factor 11
GEO: Gene Expression Omnibus
MSCs: Mesenchymal Stem Cells
MmC: Mitomycin C
RNA-seq: RNA-sequencing
